# Urinary Pregnanediol-3-Glucuronide and Pregnancy Outcomes in Frozen Embryo Transfer Cycles: A Pilot Study

**DOI:** 10.7759/cureus.83709

**Published:** 2025-05-08

**Authors:** Konstantinos Stavridis, Dimitrios Balafoutas, Stavroula-Lila Kastora, Theodoros Kalampokas, Mara Simopoulou, Ralf Joukhadar, Nikos Vlahos

**Affiliations:** 1 Department of Obstetrics and Gynaecology, Aretaieion University Hospital, Athens, GRC; 2 Faculty of Population Health Sciences, EGA (Elizabeth Garrett Anderson) Institute for Women's Health, University College London, London, GBR; 3 Department of Obstetrics and Gynaecology, University Hospital of Würzburg, Würzburg, DEU

**Keywords:** fet, luteal-phase support, pdg, pregnancy outcomes, urinary progesterone

## Abstract

Background

Frozen embryo transfers (FETs) have become more common than fresh transfers over the past decade. Progesterone levels around embryo transfer day are known to impact reproductive outcomes, albeit no clear guidelines exist regarding the optimal route, dosage, or duration of luteal phase support (LPS). A circulating progesterone threshold of about 10 ng/mL is generally accepted, but varying endometrial absorption across administration routes challenges its reliability, suggesting a need for validation through alternative progesterone measurement methods. Pregnanediol-3-glucuronide (PDG), the main urinary metabolite of progesterone, may serve as a non-invasive marker for monitoring support. This study aims to explore the association between PDG levels and pregnancy outcomes in FET cycles.

Materials and methods

This prospective pilot study was conducted at a private in vitro fertilization (IVF) center in Greece from October 2022 to May 2023. Nineteen patients undergoing FET with autologous or donor oocytes were included. Eligible participants were ≤50 years old, had a triple-layer endometrial thickness ≥6.5 mm, and received vaginal progesterone for LPS. Exclusion criteria included intrauterine anomalies, kidney disease, fresh cycles, or use of alternative endometrial preparation protocols. All patients received oral estradiol (2 mg every eight hours) for 14 days, followed by vaginal progesterone (200 mg every six hours). Spot urine samples were collected approximately 10 minutes post-transfer to assess PDG levels through ELISA (Enzyme-Linked Immunosorbent Assay). The primary outcome was the ongoing pregnancy rate (OPR); secondary outcomes included clinical pregnancy rate (CPR), biochemical pregnancy (BP), miscarriage rate (MR), and live birth rate (LBR).

Results

The median urinary PDG level was 3.5 pg/mL (interquartile range (IQR): 2.0-5.0); 21% of patients had values above 10 pg/mL, exceeding the assay’s upper detection limit. No significant associations were found between urinary PDG levels and any pregnancy outcomes (p > 0.05). A significant correlation was observed only between endometrial thickness and CPR (p < 0.05).

Conclusion

In this pilot cohort, urinary PDG levels on embryo transfer day showed no significant association with pregnancy outcomes, though the small sample size may limit conclusions. Larger studies, with standardized 24-hour urine collection, are needed to assess PDG's role in optimizing LPS in FET cycles.

## Introduction

Fresh embryo transfer cycles have been surpassed by frozen embryo transfer (FET) in fertility centers over the last 10 years [1]. The Human Fertilization and Embryology Authority reports that, while fresh transfers decreased by 8% between 2012 and 2022, the number of FET cycles more than doubled [1]. Preimplantation genetic testing for aneuploidy (PGT-A) to detect chromosomal abnormalities in embryos, improvements in vitrification and warming methods for FET cycles, and - most importantly - the markedly lower rate of ovarian hyperstimulation syndrome (OHSS), in comparison to fresh cycles, are the main causes of this change [2,3].

Enhancing pregnancy outcomes and optimizing the embryo transfer technique have been the main focus of research in recent years. Progesterone levels around the day of embryo transfer have received special attention. The literature identifies a serum progesterone threshold of approximately 10 ng/mL as one of the most widely accepted values [4,5]. While several studies underscore the detrimental effects of insufficient serum progesterone levels on pregnancy outcomes [6-8], others report that elevated circulating levels do not adversely affect outcomes [9,10]. Currently, the routes of progesterone administration (e.g., vaginal, rectal, intramuscular, and oral) and dosages vary significantly among clinicians, often guided by individual experience and resource availability. Circulating serum progesterone levels may not accurately reflect the amount of bioavailable progesterone at the endometrium. Additionally, different routes of administration may produce distinct serum progesterone thresholds due to variations in bioavailability.

To investigate these factors, this prospective pilot study aimed to evaluate urinary progesterone levels by measuring pregnanediol-3-glucuronide (PDG), the primary urinary metabolite of progesterone. The objective was to assess whether pregnancy outcomes are associated with variations in urinary PDG levels.

## Materials and methods

Study design and ethics

This prospective case-series study was conducted at a private in vitro fertilization (IVF) center in Greece (Institute of Life, Iaso) from October 2022 to May 2023. The study was approved by the Bioethics Boards of the Institute of Life, Iaso, and the National and Kapodistrian University of Athens (approval no. 480/15-12-2022). All patients included in the study signed a written informed consent form before inclusion.

Study participants

Eligible patients were women aged ≤50 years with an adequately developed endometrium (triple layer) and a thickness ≥6.5 mm, who underwent FET with either autologous or donor oocytes. Exclusion criteria included patients who refused to participate, those who underwent fresh embryo transfer, those who underwent FET with different endometrial preparation protocols than the one described below, patients with chronic or acute kidney disease, and those who experienced repeated miscarriages, had hydrosalpinx, or had intrauterine structural abnormalities such as bicornuate, unicornuate, or didelphic uterus. The demographic information gathered included the patients' age, body mass index (BMI, kg/m²), IVF indications (male, tubal, unexplained, ovulatory, and mixed), gravidity, parity, and PDG concentration (pg/mL), as well as urine specific gravity and endometrial thickness (mm) prior to progesterone introduction. The number of embryos transferred, the stage of the transferred embryo (cleavage or blastocyst), the positive pregnancy rate (serum beta human chorionic gonadotropin (hCG) >25 IU/L per transfer), the clinical pregnancy rate (CPR) (fetal heartbeat by transvaginal ultrasound), the miscarriage rate (MR) (any clinical pregnancy lost before pregnancy week 12), the ongoing pregnancy rate (OPR) at 12 weeks of pregnancy, and the live birth rate (LBR) were also gathered.

Endometrial preparation and embryo transfer technique

For endometrial preparation, all patients received 2 mg of oral estradiol (E2) valerate (Cyclacur) every eight hours for 14 days. Endometrial thickness was assessed using 2D transvaginal ultrasound, and once the appropriate thickness was confirmed, vaginal progesterone capsules (200 mg every six hours, Utrogestan) were initiated. This protocol followed the institution’s standard practice and was continued until 10-12 weeks of gestation. Embryo transfer was performed on day 3 or day 5, depending on the developmental stage of the embryo (cleavage or blastocyst). Embryo quality was assessed using morphological criteria as follows: cleavage-stage embryos were classified as good, fair, or poor based on cell number (optimal: 6-8 cells on day 3), fragmentation (<10% for good), and blastomere symmetry. Blastocysts were graded by expansion, inner cell mass (ICM), and trophectoderm (TE); grades ≥3BB were considered good, 3BC or 4CB as fair, and any with grade C in ICM or TE as poor quality. Vitrification and warming were performed using commercial vitrification kits (Kitazato, Los Angeles, CA, USA). All embryo transfers were conducted under transabdominal ultrasound guidance with the patient having a moderately full bladder. Transfers were performed by an experienced gynecologist using a Wallace 18 cm catheter.

PDG evaluation

Spot urine samples for the evaluation of the progesterone metabolite PDG levels were collected from all patients on the day of embryo transfer, approximately 10 minutes post-transfer. Urine samples were then analyzed using enzyme immunoassays (Arbor Assays, Ann Arbor, MI, USA). For samples to be within the linear range of the ELISA (Enzyme-Linked Immunosorbent Assay), they needed to be heavily diluted. The final readings, expressed in pg/mL, were then multiplied by the dilution factor in order to determine the initial concentrations.

Outcomes

The primary outcomes examined included OPRs. Secondary outcomes were biochemical pregnancy (BP), MR, CPR, and LBRs.

Statistical analysis

Continuous variables are presented as medians with interquartile ranges (IQRs), whereas categorical variables are summarized as frequencies with corresponding percentages. Dependent variables were the primary and secondary outcomes, while independent variables were PDG at embryo transfer, patient age, BMI, endometrial thickness when receiving estrogen, and transfer distance from the fundus.

The Mann-Whitney test was employed to explore intergroup differences of numerical variables, considering non-parametric distribution given the nature of the study. A p-value ≤0.05 was considered statistically significant. The correlation matrix, with computed Pearson R² and p-values of comparisons, was computed across dependent and independent variables. Statistical analysis was performed using GraphPad Prism V10 (GraphPad Software, San Diego, CA, USA) under an academic license.

## Results

In our cohort of 19 patients undergoing FET cycles, baseline characteristics are summarized in Table 1. The median age was 36 years (IQR: 33-40 years), with a median BMI of 23.4 kg/m² (IQR: 21.5-25.8 kg/m²). The indications for IVF were heterogeneous, with polycystic ovary syndrome (PCOS) observed in 21% of cases, tubal factor infertility in 32%, male factor in 16%, unexplained infertility in 26%, and a mixed etiology in 5%. Median gravidity and parity were 2 (IQR: 1-3) and 1 (IQR: 0-2), respectively. Endometrial thickness at the time of estrogen administration was 8.0 mm (IQR: 7.0-9.0 mm). While most patients received two embryos (94.7%), one patient received a single embryo. Embryo stage at transfer was split between day 3 (37%) and day 5 (63%), and the quality of embryos was predominantly classified as good (63%), followed by fair (26%) and poor (11%). The median transfer distance from the fundus was 10 mm (IQR: 8-12 mm).

**Table 1:** Patient characteristics *Numeric values only IQR, interquartile range; BMI, body mass index; IVF, in vitro fertilization; PCOS, polycystic ovary syndrome; PDG, pregnanediol-3-glucuronide

Parameter (Measurement Unit)	Value (Median (IQR) or n%)
Age (Years)	36 (33-40)
BMI (kg/m²)	23.4 (21.5-25.8)
IVF Indication	PCOS: 4 (21%); Tubal: 6 (32%); Male Factor: 3 (16%); Unexplained: 5 (26%); Mixed: 1 (5%)
Gravidity	2 (1-3)
Parity	1 (0-2)
Endometrial Thickness (mm)	8.0 (7.0-9.0)
Number of Transferred Embryos	1 embryo: 1 (5.3%); 2 embryos: 18 (94.7%)
Embryo Stage at Transfer	Day 3: 7 (37%); Day 5: 12 (63%)
Quality of Embryos	Good: 12 (63%); Fair: 5 (26%); Poor: 2 (11%)
Transfer Distance From Fundus (mm)	10 (8-12)
PDG at Embryo Transfer Day (pg/mL)	3.5 (2.0-5.0)*; 4 (21%) had “>10”

**Figure 1:**
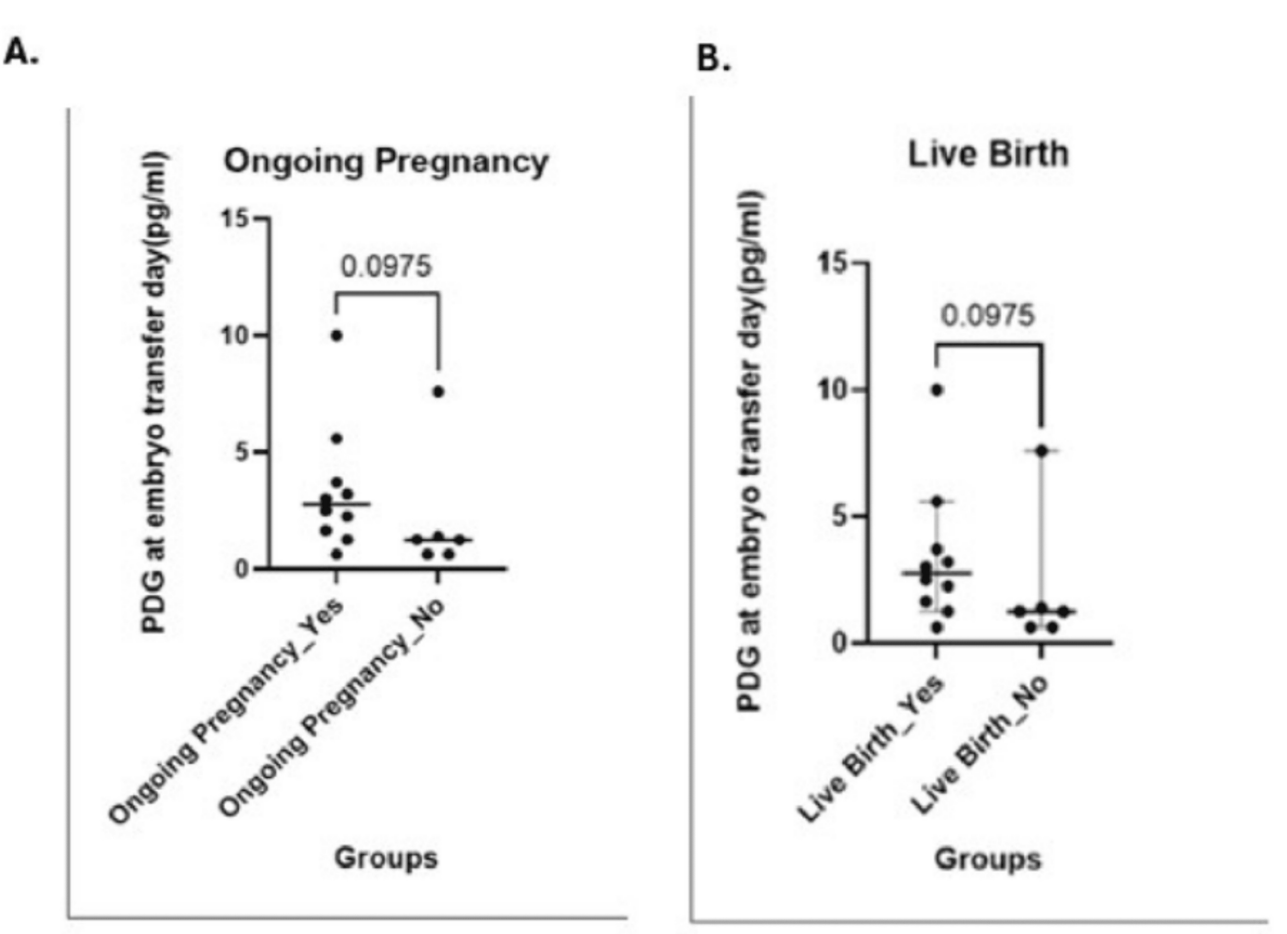
(A) Ongoing pregnancy rates (p-value: 0.09, two-tailed; Difference Hodges-Lehmann: -1.430); (B) Live birth rates (p-value: 0.09, two-tailed; Difference Hodges-Lehmann: -1.430) PDG, pregnanediol-3-glucuronide

**Figure 2:**
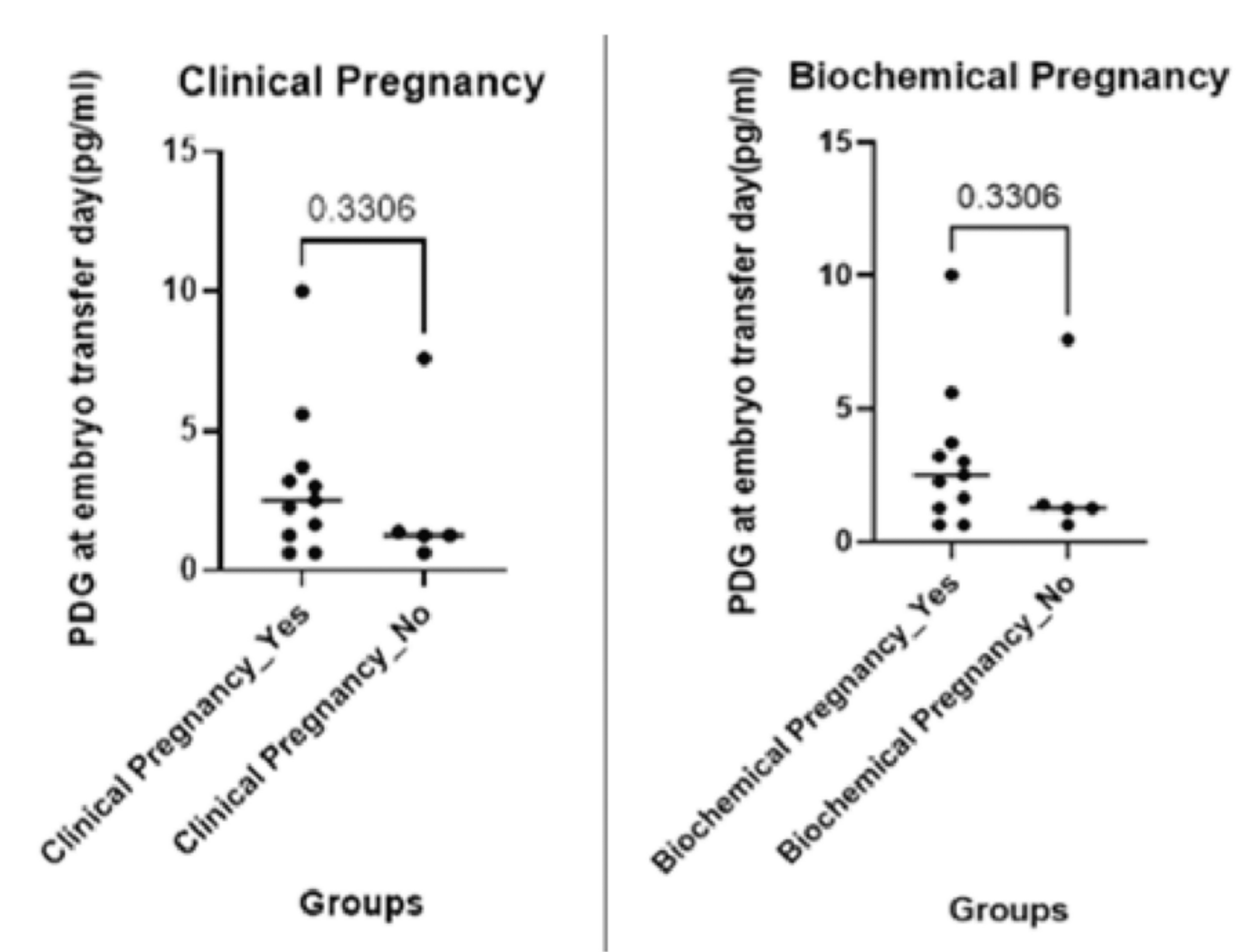
(A) Clinical pregnancy rates (p-value: 0.33, two-tailed; Difference Hodges-Lehmann: -1.430); (B) Biochemical pregnancy rates (p-value: 0.33, two-tailed; Difference Hodges-Lehmann: -1.010) PDG, pregnanediol-3-glucuronide

**Figure 3:**
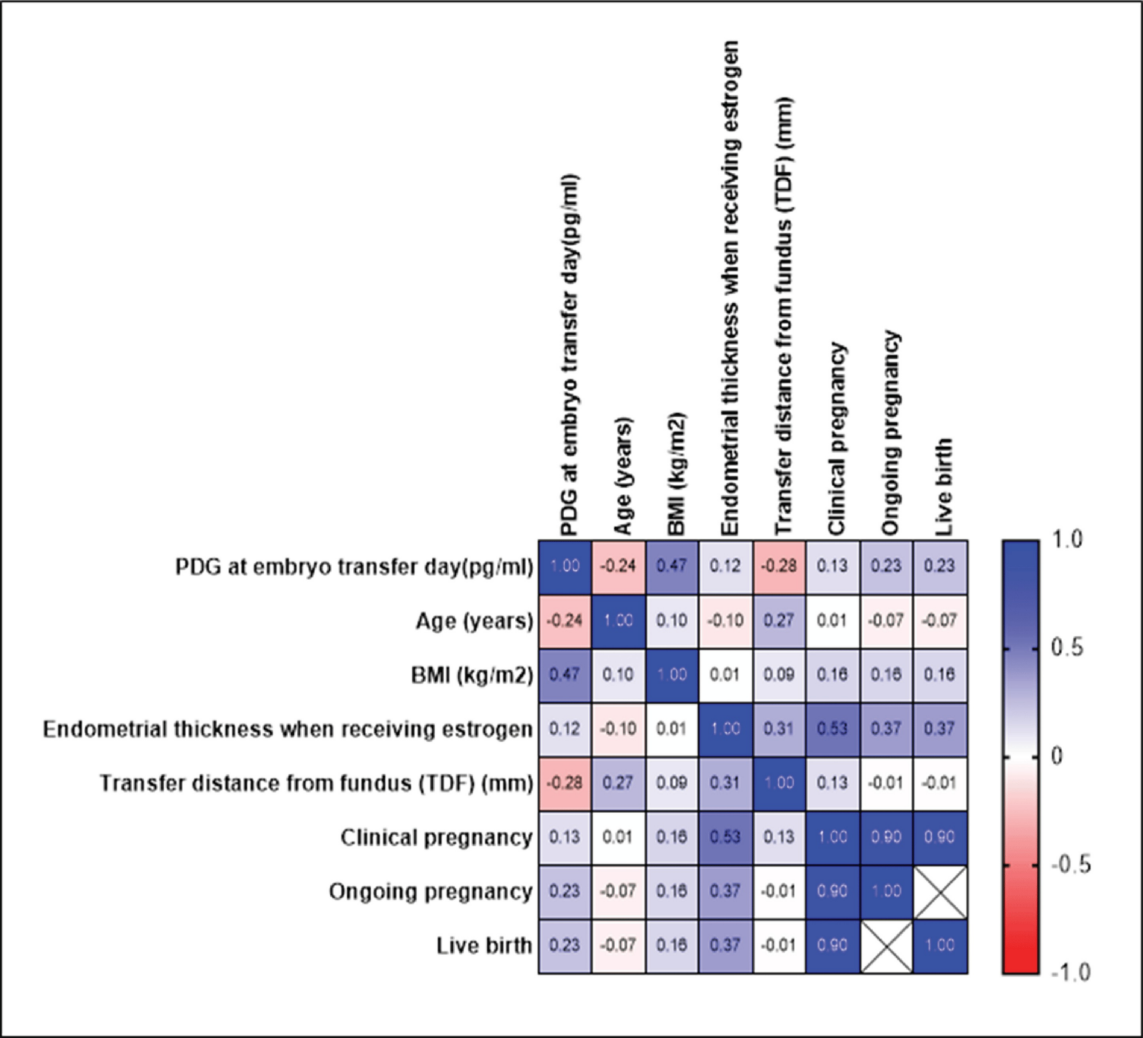
Heatmap image of correlation coefficients Heatmap of correlation coefficients among PDG levels on embryo transfer day (pg/mL), patient characteristics (age, BMI), endometrial thickness (during estrogen treatment), TDF, and reproductive outcomes (clinical pregnancy, ongoing pregnancy, live birth). Positive correlations are shown in red, negative correlations in blue, and the color intensity reflects the magnitude of the correlation coefficient. PDG, pregnanediol-3-glucuronide; BMI, body mass index; TDF, transfer distance from fundus

For PDG levels on the day of embryo transfer, numeric entries yielded a median of 3.5 pg/mL (IQR: 2.0-5.0 pg/mL); however, 21% of patients had PDG values reported as >10 pg/mL, indicating levels above the assay’s upper limit. In our cohort of 19 patients, 12 (63.2%) had a positive hCG, 12 (63.2%) achieved a clinical pregnancy, 11 (57.9%) had an ongoing pregnancy, and 11 (57.9%) resulted in a live birth; miscarriage occurred in one patient (5.3%). The analysis revealed that urinary PDG levels were not significantly associated with any of these reproductive outcomes (p > 0.05), except for a significant association observed between endometrial thickness and CPRs (p < 0.05); however, no specific threshold value could be identified, as the relationship appears to be linear rather than indicative of a clear clinical cut-off. Future studies may help determine whether a clinically meaningful cut-off exists. Additionally, due to the low occurrence of miscarriage events (1/19), no formal statistical test was conducted for this outcome. These findings indicate that, within our cohort, urinary PDG levels do not appear to influence the measured reproductive outcomes (Figures 1-3).

## Discussion

This study aims to investigate the potential association between pregnancy rates and PDG levels in urine in patients undergoing FET cycles. The results highlighted no statistically significant differences in the median PDG levels among the different pregnancy outcomes - namely, ongoing pregnancies, live births, miscarriages, clinical pregnancies, and positive pregnancy tests.

Currently, there are no specific guidelines regarding the route, dosage, or duration of progesterone administration during IVF cycles. Recent studies, however, have placed a lot of emphasis on the association between progesterone levels and pregnancy outcomes. In a study by Lightman et al. in 1999 [11], intramuscular and vaginal routes of progesterone administration were compared. The researchers reported that the vaginal strategy was linked to higher pregnancy rates, albeit the intramuscular approach resulted in higher serum progesterone concentrations. In terms of reproductive outcomes, a later randomized trial found that intramuscular injections are non-inferior to the vaginal approach [12]. Furthermore, a large prospective study examined the effectiveness of vaginal progesterone alone versus vaginal progesterone plus oral dydrogesterone in supporting the luteal phase. According to the study, adding dydrogesterone to vaginal progesterone greatly decreased the rate of miscarriages and enhanced the outcomes of live births [13].

Despite the lack of specific guidelines supporting its use, vaginal progesterone has become more and more prominent in recent years as a means of supporting the luteal phase in FET cycles. The vaginal method has been demonstrated to offer superior endometrial absorption and a more localized effect because of its uterine first-pass effect [14]. However, it also results in lower serum concentration levels due to a shorter elimination half-life compared to other administration routes [15]. Determining a specific threshold below which pregnancy rates decrease is challenging, because different progesterone administration methods produce different serum progesterone concentrations. The various progesterone regimens that clinicians use further exacerbate this variability. According to a thorough meta-analysis by Melo et al., patients with serum progesterone levels below the cutoff had lower pregnancy rates in studies that reported a threshold of 10 ng/mL. Nevertheless, different studies reported different thresholds; some identified lower or higher values based on their clinical experience and analyses [4]. This raises the important question of whether circulating progesterone levels accurately reflect the bioavailable progesterone for the endometrium. Additionally, the vaginal, intramuscular, subcutaneous, and oral routes of administration must be evaluated to determine if they can be reliably compared or combined to establish a universally accepted threshold. Variations in clinical practices and differences in the bioavailability of progesterone across routes contribute to significant uncertainty regarding the reliability of serum progesterone measurements. Validation through alternative methods, such as urine progesterone measurements, could provide a promising solution.

To our knowledge, this is the first study to assess the association between urinary PDG levels and pregnancy outcomes in FET cycles, as no similar studies focusing on FET cycles have been reported in the existing literature. Although PDG is a well-established marker for ovulation, with levels greater than 5 μg/mL considered confirmatory [16,17], our subjects - who received high doses of exogenous progesterone for luteal phase support (LPS) - exhibited substantially elevated PDG concentrations in urine. Consequently, samples required extensive dilution to fall within the ELISA's linear range, and the final measurements, expressed in pg/mL, were multiplied by the dilution factor to estimate the original concentrations. This methodological difference accounts for the discrepancy in reported units between our study and previous reports.

Due to the variability in serum progesterone levels, influenced by factors such as diurnal fluctuations and differing administration methods, urinary PDG measurement may serve as a more stable and non-invasive tool for determining progesterone thresholds associated with IVF success in frozen cycles. However, the limited sample size of our pilot study, consisting of 19 participants, reduced the statistical power, potentially obscuring meaningful associations and precluding multivariate analysis of confounding factors. The absence of significant findings may be attributed to this constraint, rather than a true lack of effect. This highlights the need for larger-scale studies to further explore and validate these observations. A key limitation of our approach was the use of spot urine samples, which are subject to intra-day variability and may not fully capture cumulative hormone exposure. As such, a 24-hour urine sample may offer a more stable measure of PDG than the spot samples used in our study. Larger studies employing 24-hour collections are needed to define accurate PDG thresholds on embryo transfer day, after which simple test strips could be evaluated as a supplementary tool alongside serum progesterone measurements.

## Conclusions

Our pilot study evaluated the association between urinary PDG levels and reproductive outcomes in FET cycles. We found no statistically significant differences between urinary PDG levels and ongoing pregnancy, live birth, BP, or MRs. These findings suggest that, within the range observed in our cohort, urinary PDG levels do not reliably predict reproductive outcomes. However, given the limited sample size of this pilot study, further research in larger cohorts is necessary to validate these observations and to explore the potential of urinary PDG as an alternative marker for optimizing LPS in FET cycles.
